# Predictive modeling in pediatric traumatic brain injury using machine learning

**DOI:** 10.1186/s12874-015-0015-0

**Published:** 2015-03-17

**Authors:** Shu-Ling Chong, Nan Liu, Sylvaine Barbier, Marcus Eng Hock Ong

**Affiliations:** Department of Emergency Medicine, KK Women’s and Children’s Hospital, Singapore, Singapore; Department of Emergency Medicine, Singapore General Hospital, Singapore, Singapore; Centre for Quantitative Medicine, Duke-NUS Graduate Medical School, Singapore, Singapore; Health Services and Systems Research, Duke-NUS Graduate Medical School, Singapore, Singapore

**Keywords:** Brain injuries, Child, Prediction rules, Machine learning

## Abstract

**Background:**

Pediatric traumatic brain injury (TBI) constitutes a significant burden and diagnostic challenge in the emergency department (ED). While large North American research networks have derived clinical prediction rules for the head injured child, these may not be generalizable to practices in countries with traditionally low rates of computed tomography (CT). We aim to study predictors for moderate to severe TBI in our ED population aged < 16 years.

**Methods:**

This was a retrospective case–control study based on data from a prospective surveillance head injury database. Cases were included if patients presented from 2006 to 2014, with moderate to severe TBI. Controls were age-matched head injured children from the registry, obtained in a 4 control: 1 case ratio. These children remained well on diagnosis and follow up. Demographics, history, and physical examination findings were analyzed and patients followed up for the clinical course and outcome measures of death and neurosurgical intervention. To predict moderate to severe TBI, we built a machine learning (ML) model and a multivariable logistic regression model and compared their performances by means of Receiver Operating Characteristic (ROC) analysis.

**Results:**

There were 39 cases and 156 age-matched controls. The following 4 predictors remained statistically significant after multivariable analysis: Involvement in road traffic accident, a history of loss of consciousness, vomiting and signs of base of skull fracture. The logistic regression model was created with these 4 variables while the ML model was built with 3 extra variables, namely the presence of seizure, confusion and clinical signs of skull fracture. At the optimal cutoff scores, the ML method improved upon the logistic regression method with respect to the area under the ROC curve (0.98 vs 0.93), sensitivity (94.9% vs 82.1%), specificity (97.4% vs 92.3%), PPV (90.2% vs 72.7%), and NPV (98.7% vs 95.4%).

**Conclusions:**

In this study, we demonstrated the feasibility of using machine learning as a tool to predict moderate to severe TBI. If validated on a large scale, the ML method has the potential not only to guide discretionary use of CT, but also a more careful selection of head injured children who warrant closer monitoring in the hospital.

## Background

Head Injury remains an important cause of mortality and morbidity for children, worldwide. Injury-related deaths in the pediatric age group are mostly associated with head injury [1]. Emergency Departments (EDs) worldwide are seeing an increase in pediatric head injury attendance [2]. The admission rates for head injured children are also on the rise [3]. While the majority of these are mild, severe head injuries have potential for mortality and long-term neurological devastation. The prevalence of neurological disability among children and youths admitted for traumatic brain injury approximates 20% [4]. Compared to adults with head injury, children tend to present in a varied way. Younger children are unable to provide a clear history and may be difficult to examine. A matched retrospective cohort study performed to inform an evidence-based triage assessment showed that young age and injuries to the temporo-parietal region were more likely to be associated with significant closed head injury, as identified on computed tomography (CT) [5].

CT scans are frequently performed in the adult head injured population. In children however, the rapidly developing brain, when exposed to radiation, is at risk of developing malignancies [6,7]. When deciding on whether a CT is warranted in a young child, the physician has to weigh the need to promptly diagnose an intracranial injury against the radiation that the child will be exposed to. Locally, there is great reluctance to order unnecessary CT scans.

Clinical prediction rules [8-10] have been published by large North American research networks to guide the ED physician on when to order a CT scan for a head-injured child. The Pediatric Emergency Care Applied Research Network (PECARN) [7] rule specifically, has been reported to be of excellent performance [11]. However, prior to application, it has been encouraged that the question of generalizability and performance to the individual population be addressed [12]. The CT rate in the Singapore population has been maintained at a low level of under 2%, as opposed to the estimated 30-50% reported in the literature. This is because a large majority of our patients comprise of young children presenting with mild head injuries after falls, as well as the availability of inpatient observation in most cases.

While most of the published clinical rules [6-8] were derived with recursive partitioning [13], emerging computational methods like machine learning (ML) have potential in solving complex and challenging medical problems [14-17]. ML procedures are capable of discovering interaction, nonlinear, and high-order effects in the predictive variables [14], which are difficult to handle with conventional parametric regression methods. In this study, we aim to (1) select clinical predictors for moderate to severe traumatic brain injury (TBI) in children aged < 16 years, (2) derive a ML model and a logistic regression model (3) Compare the performance of both tools.

## Methods

### Study design and patient recruitment

This was a retrospective case–control study. Cases were included if patients presented during the period from 2006 to 2014, with moderate to severe TBI. Due to the very low event rate, a case–control design was chosen [18,19] instead of a cohort analysis.

Data was collected from KK Women’s and Children’s Hospital, Singapore, the main pediatric emergency department in Singapore, with an annual trauma attendance (of all severities) of about 28,000. The majority of head injuries that we see in the emergency department are mild. We defined cases as patients aged < 16 years who presented to the ED with a Glasgow coma scale (GCS) of ≤13 or those who presented with GCS 15 but deteriorated after admission, and were confirmed on CT scan to have a bleed or fracture, during the period January 2006 – June 2014. Controls were obtained from an ongoing prospective head injury database. Controls were age-matched, year for year, at a ratio of 4 controls: 1 case. This study was approved by the Singapore Health Services (SingHealth) Centralized Institutional Review Board with a waiver of patient consent.

We obtained the individual predictive variables based on those published in similar studies [6-8], as well as from departmental head injury protocols. We divided the collection of data into demographics, mechanism of injury, presenting symptoms and physical examination findings. Symptoms studied included seizures, confusion, loss of consciousness (and duration), difficult arousal, and vomiting. Caregivers of preverbal children were questioned for irritability while verbal children were questioned for headache and amnesia.

From the physical findings, data were documented on the GCS, altered mental status, presence of unequal pupils, signs of vault fractures and basal skull fractures, scalp hematoma, focal neurological signs and gait abnormalities. Basal skull fractures signs included: blood or cerebrospinal fluid from the nose or ears, bruising at the posterior auricular region, and periorbital bruises. Among young children with open fontanelles, the presence or absence of a tense fontanelle was documented.

Admitted patients were followed up and the need for neurosurgery or any resultant death was documented. Among the controls, a clinical research coordinator followed up discharged patients with a call 72 hours after ED attendance, to ask about any deterioration or attendance at another institution.

### Statistical analysis

Patients with and without TBI were studied for differences in clinical characteristics, using Student t-tests for continuous variables and Chi-Square or Fisher Exact test for categorical variables. Continuous variables are expressed as mean and standard deviation and categorical variables as absolute numbers and percentages.

In the approach using a classical logistic regression, we used a two-step selection for the contributing factors. Univariable logistic regressions were performed on each of them and those achieving a p-value below 0.2 were selected. Then, we fitted a multivariable model, following a stepwise algorithm (p-value of entry = 0.1, p-value of removal = 0.05). The models’ selection was based on the Akaike Criterion (AIC), the Bayesian Information Criteron (BIC) and log-likelihood, incorporating clinical knowledge. The predictive performance of the final model was reflected by the AUC, sensitivity, specificity, Positive and Negative Predictive Values (PPV and NPV). The data were analyzed using STATA v12 (Stata Corp, College Station, TX) and MATLAB R2009a (Mathworks, Natick, MA).

### Predictive modeling with machine learning

The machine learning (ML) method [20] implemented for predictive modeling in this pediatric traumatic brain injury study was originally designed for the prediction of acute cardiac complications, with an ensemble learning-based risk assessment as the core of decision making. The rationale behind this ML method is that in most scenarios we often seek a second or more opinion before making final decisions. For example in choosing a proper treatment of a disease, people usually consult with more than one physician to reach a conclusion. In machine learning, this process of decision making is called ensemble learning where the decision is made by combining the outcomes of several individual classifiers (a classifier in machine learning is considered as a physician in the real-world).

**Figure 1:**
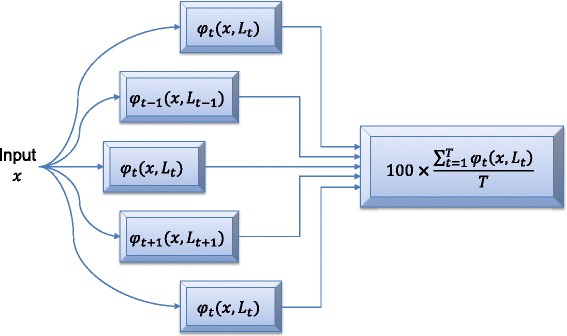
**The architecture of the machine learning (ML) method.** Input *x* is the patient whose risk of abnormal CT scan is being evaluated. *L*_*t*_ is the training set consisting of *K* samples (*x*_*k*_, *y*_*k*_) where *k* = 1, 2, …, *K* and *y*_*k*_ is the class label. By using the training data, a total of *T* individual classifiers *φ*_*t*_(*x*, *L*_*t*_) are created to form the decision ensemble. Each individual classifier is built based on a subset of the training data. Then the prediction outcomes are combined by means of majority voting scheme to generate a final risk score for patient *x*.

Due to its flexibility in many application domains, the above ML method is readily adaptable to our study with minor changes. The architecture of the ML method used in this study is illustrated in Figure 1. Each ensemble classifier *φ*_*t*_ where *t* = 1, 2, …, *T* and *T* is the number of individual classifiers in the decision ensemble. Ensemble learning methods [21,22] usually generate a predictive label rather than a score as the output. The ML method uses a simple and straightforward approach to convert the predictive decision into a risk score. Details are elaborated as follows.

Assume that we have a training dataset *L*_*t*_ consisting of *K* samples (*x*_*k*_, *y*_*k*_) where *k* = 1, 2, …, *K* and *y*_*k*_ is the class label. Given a testing sample *x*, its label *y* can be predicted by a single classifier *φ*_*t*_(*x*, *L*_*t*_) where the class label is either *C*_0_ or *C*_1_. Label *C*_0_ indicates that the patient is normal (negative outcome) while label *C*_1_ indicates that the patient has abnormal CT scan (positive outcome). As illustrated in Figure 1, we can derive *T* independent classifiers from training samples. The risk score on the testing sample *x* is calculated using

1 $$ R{S}_x=100\times \frac{{\displaystyle {\sum}_{t=1}^T{\varphi}_t\left(x,{L}_t\right)}}{T} $$

The advantage of the ML method is its ability to handle data imbalance, select suitable individual classifiers for decision ensemble creation and decision combination, such as for our dataset (i.e. positive samples are less than negative samples with a ratio of 1:4).

Instead of applying a sophisticated hybrid-sampling scheme [20] to create the decision ensemble, in this study we used a simplified under-sampling scheme. Given the minority set *P* and the majority set *N*, the under-sampling method [21] randomly samples a subset *N*_*t*_ from *N* where |*N*_*t*_| < |*N*| and |*N*_*t*_| = |*P*|. Dataset *P* represents a set of samples with positive outcomes and *N* represents a set of samples with negative outcomes. The balanced dataset *L*_*t*_ consists of both *P* and *N*_*t*_ and is used for classification model derivation. We then estimate a risk score using Eq. (1).

In the ML method, neural network [23,24] was chosen as the individual classifier *φ* because of its reliable performance and efficiency. The individual classifier was single layer feed-forward neural network where extreme learning machine [25] was adopted as the training method. In implementing the ensemble learning and neural network-based risk scoring method, the ensemble size *T* was 100, and the number of hidden neurons was 30. The sigmoid function was chosen as the activation function in neural network training.

In our study, two sets of predictive variables were used to build the ML model. One set of variables was derived from logistic regression according to the statistical significance, while another set of variables were determined by physicians in terms of clinically relevance. Compared to traditional regression analysis, the ML method is flexible where the predictive variables used to build the model are not necessarily significant in statistical analysis. Furthermore, the ML method may be able to discover nonlinear correlations among all variables.

## Results

Thirty-nine cases of moderate to severe TBI children were analyzed, with a corresponding 156 age-matched controls. Table 1 shows the comparison of patient demographics and mechanism of injury, between both groups. Among the cases, 26 patients required neurosurgical intervention and 8 patients died. From the prospective database, our event rate was 0.5% and our CT rate was 1%. Among the controls in this study, 4 patients had a CT brain (2.6%). Retrospective application of the published rules [6-8] to the prospective database showed that they would indeed increase the CT rate in our population: CHALICE 24.0%, CATCH (for high risk only) 5.7%, CATCH (for high and medium risk) 20.1%, PECARN (for high risk in children < 2 years) 1.7%, PECARN (for high risk in children ≥ 2 years) 2.1%, PECARN (high and intermediate risk in children < 2 years) 14.0%, PECARN (high and intermediate risk in children ≥ 2 years) 24.6%.

**Table 1:** **Patient demographics and mechanism of injury**

	**Traumatic brain injury (Cases) N = 39**	**No traumatic brain injury (Controls) N = 156**	**p-value**
Age mean (SD)	8.11 (4.25)	8.10 (4.21)	0.99^1^
Female (%)	14 (36%)	44 (28%)	0.347^2^
Primary mechanism			<0.001^2^
Fall (%)	19 (49%)	110 (70%)	
Road traffic accident (%)	17 (44%)	3 (2%)	
Struck by projectile (%)	0 (0%)	3 (2%)	
Non-accidental injury (%)	2 (5%)	9 (6%)	
Others (%)	1 (2%)	31 (20%)	
Primary mechanism among children ≤ 2 years old (N = 5 vs 20)			0.012^2^
Fall (%)	3 (60%)	18 (90%)	
Road traffic accident (%)	2 (40%)	0 (0%)	
Struck by projectile (%)	0 (0%)	0 (0%)	
Non-accidental injury (%)	0 (0%)	0 (0%)	
Others (%)	0 (0%)	2 (10%)	

^1^Student t-test ^2^Chi-Square test.

Table 1 presents patient demographics. With regards to the primary mechanism of injury, 44% of the cases were involved in a road traffic accident as compared to only 2% in the controls (p < 0.001), while the majority of controls presented to the ED after falls. A similar trend was seen (although with small numbers) among children aged 2 years and under.

Table 2 describes the individual variables obtained from history and physical examination. Variables from history or physical evidence that described altered mental status – difficult arousal, confusion/disorientation and signs of altered mental status were each statistically significant. Besides altered mental status, the presence of signs of base of skull fracture, unequal pupils, and scalp hematoma were statistically significant. Among those with scalp hematomas, frontal hematomas appeared to be protective. Among infants with open fontanelles, the presence of a tense fontanelle was also statistically significant.

**Table 2:** **Univariable analysis of variables from history and physical examination**

	**Traumatic brain injury (Cases) N = 39**	**No traumatic brain injury (Controls) N = 156**	p-value*
**History**			
Loss of consciousness	25 (64%)	8 (5%)	<0.001
If Yes, then number who lost consciousness for > 1 minute (%)	24 (96%)	4 (50%)	<0.001
Difficult arousal (%)	27 (70%)	3 (2%)	<0.001
Vomiting (%)	11 (28%)	26 (17%)	0.113
Seizure activity (%)	6 (15%)	0 (0%)	<0.001
Confusion/Disorientation (%)	33 (85%)	3 (2%)	<0.001
(Preverbal) irritability (%) (N = 7 vs 22)	0 (0%)	1 (5%)	1
(Verbal) Headache (%) (N = 32 vs 130)	8 (25%)	45 (35%)	0.401
(Verbal) Amnesia (%) (N = 32 vs 131)	1 (3%)	6 (5%)	1
**Physical examination**			
Signs of altered mental status (%)	36 (95%)	1 (1%)	<0.001
Presence of unequal pupils (%)	(26%)	(1%)	<0.001
Clinical signs of skull fracture (%)	2 (5%)	1 (1%)	0.103
Signs of base of skull fracture (%)	13 (33%)	4 (3%)	<0.001
Presence of scalp hematoma (%)	20 (51%)	36 (23%)	<0.001
Frontal (%) (N = 20 vs 36)	1 (5%)	13 (36%)	0.011
Presence of scalp laceration	7 (18%)	32 (21%)	0.825
(Preverbal) with open fontanelle (N = 7 vs 22)	4 (57%)	17 (77%)	0.357
Presence of tense fontanelle among those with open fontanelles (N = 4 vs 17)	3 (75%)	0 (0%)	0.03

*Chi-Square or Fisher Test when appropriate.

On multivariable analysis (Table 3), the following four predictors showed an independent significant effect: mechanism of road traffic accident (OR: 19.62, p = 0.001), history of loss of consciousness (OR: 16.32, p < 0.001), vomiting (OR: 4.89, p = 0.006) and signs of base of skull fracture (OR: 13.94, p = 0.001). A ML model was created using three more variables, namely presence of seizure activity, confusion and clinical signs of skull fracture.

**Table 3:** **Independent predictors for traumatic brain injury (univariable then multivariable logistic regressions)**

**Predictor**	**Adjusted OR [95% CI]**	**p-value**
Road traffic accident	19.62 [3.61-106.66]	0.001
History of loss of consciousness	16.32 [4.95-53.76]	<0.001
Vomiting	4.89 [1.57-15.27]	0.006
Signs of base of skull fracture	13.94 [2.74-70.86]	0.001

**Figure 2:**
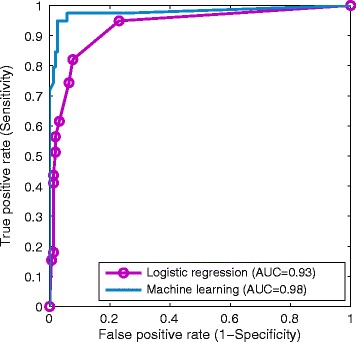
**ROC curves produced by logistic regression and machine learning.**

Two receiver operating characteristic (ROC) curves shown in Figure 2 were drawn using both prediction models, from which ML method was observed to outperform logistic regression method. Detailed comparison results are presented in Table 4. In general, the ML method significantly improved upon the logistic regression method with respect to sensitivity (94.9% vs 82.1%) and PPV (90.2% vs 72.7%). The cutoff scores were chosen to give the best trade-off between sensitivity and specificity, where the optimal cutoff is determined by the point that is nearest to the upper-left corner in the ROC curve.

**Table 4:** **Prediction results using receiver operating characteristic (ROC) analysis**

	**Machine learning** ^**1,2**^	**Logistic regression** ^**3,4**^
**AUC [95% CI]**	0.98 [0.95-1]	0.93 [0.87-0.99]
**Cutoff score**	49	0.25
**Sensitivity [95% CI]**	94.9% [87.9%-100%]	82.1% [70.0%-94.1%]
**Specificity [95% CI]**	97.4% [95.0%-99.9%]	92.3% [88.1%-96.5%]
**PPV [95% CI]**	90.2% [81.2%-99.3%]	72.7% [59.6%-85.9%]
**NPV [95% CI]**	98.7% [96.9%-100%]	95.4% [92.0%-98.7%]

AUC: area under the curve; CI: confidence interval; PPV: positive predictive value; NPV: negative predictive value.

^1^The range of machine learning score is [0, 100].

^2^Variables used in the machine learning method were road traffic accident, history of loss of consciousness, vomiting, seizure activity, confusion, clinical signs of skull fracture, and signs of base of skull fracture.

^3^The range of logistic regression score is [0, 1].

^4^Variables used in the logistic regression model were road traffic accident, history of loss of consciousness, vomiting, and signs of base of skull fracture.

**Figure 3:**
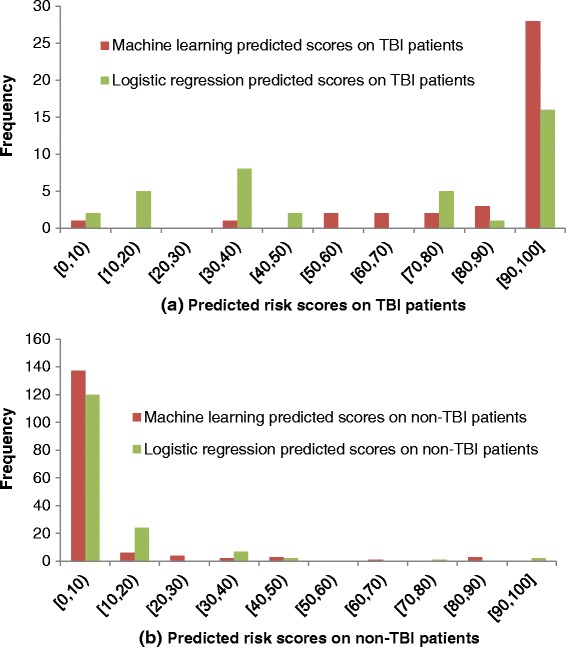
**Frequency distribution of the logistic regression method and the machine learning method in predicting pediatric TBI.**

Figure 3 illustrates the differences in predicted scores by the logistic regression method and the ML method in terms of frequency distribution. Figure 3(a) shows the results on TBI patients and Figure 3(b) presents the results on non-TBI patients. In non-TBI patients, both methods performed similarly with the ML prediction being slightly more accurate. In TBI patients, the ML method performed better at categorizing most of the TBI patients at high risk for moderate to severe injury. These matched the observations that the ML method achieved higher sensitivity and PPV than the logistic regression method.

## Discussion

In current practice, 3 clinical decision rules (CDRs) have been widely referenced: CHALICE, PECARN and CATCH. PECARN and physician practice were demonstrated to be superior in identifying all clinically important traumatic brain injuries in a recent prospective observational study that compared these rules [11]. Specifically, apart from being derived and validated in a large population (n = 42412), the PECARN had a separate rule for preverbal children (<2 years old) [7]. The PECARN was intended as a rule-out tool, identifying low risk children who do not require the CT scan. The rate of CT in this study was 35.3%.

It has been previously noted that applicability of the clinical prediction rules may vary based on population characteristics, and before implementing them, their performance should first be assessed [10]. We identified a few differences in the Singapore population compared to that reported in the PECARN study. The mean age of children from our prospective database was 4.6 years, as compared to 7.1 years in the latter. Most of our head injured population comprised of young children presented after low mechanism falls. This likely accounted for the low event rate in our population – a unique characteristic that may hinder the direct use of the above clinical decision rules. Our center sees a low event rate of moderate to severe TBI (<1%) and a baseline CT rate of less than 2%. We found that the direct application of these rules to our population would in most cases, increase our CT rate, which would be undesirable. Hence, we sought to derive high risk clinical predictors from our population, and test their utility in our local setting.

The multivariable analysis revealed 4 independent predictors – road traffic accident as the mechanism of injury, a history of loss of consciousness, vomiting and signs of base of skull fracture. The presence of a change in conscious level and evidence of base of skull fracture were consistently reported in the 3 high performing CDRs. The presence of vomiting, on the other hand, was variable (reported in PECARN for children 2 years and older, as well as 3 or more discrete episodes of vomiting in CHALICE). Dayan et al., on the other hand, reported that the presence of isolated vomiting among children with a minor blunt head injury was unlikely to be associated with clinically important TBI [26].

We also investigated the utility of ML for predicting pediatric TBI. Compared with the logistic regression method, ML is more flexible in terms of predictor selection as it is able to discover nonlinear interactions among clinical variables [14]. As a result, the presence of seizure activity, confusion and clinical signs of skull fracture were combined with the above mentioned four variables used in regression method to build a ML predictive model. It is observed in Table 2 that both seizure activity and confusion are statistically significant, while the presence of clinical signs of skull fracture is not. Possible explanations on improved performance by adding in non-significant variables are that a complex neural network structure is capable of detecting nonlinear correlations among variables and associating them with the clinical outcome, i.e. TBI in our study. There is superiority of ML over logistic regression as shown in Figure 2 and Table 4 where at the optimal cutoff scores ML achieves much higher sensitivity and PPV. However, it is worth noting that all reported performance indicators have overlapping confidence intervals. Further investigation will be conducted to determine if the ML method is statistically superior to classic logistic regression method.

To the best of our knowledge, machine learning has yet been applied to predict pediatric TBI, although it received attention in various medical areas [14,15,20,27]. Amongst many machine learning methods, neural network has been widely implemented for predictive modeling and shows excellent prediction performance compared to logistic regression [28-31]. The ability of a neural network to model complex nonlinear relationships between independent and dependent variables [32] makes it a natural tool to predict moderate to severe TBI in our study. However the application of neural networks is limited by the lack of interpretability, more specifically, the difficulty in assessing the relative contribution of each variable to the predictive modeling [31]. In developing predictive models, it is usually recommended to consider both advantages and limitations of the approaches [32,33].

We believe that our findings may apply to populations with low event rates of moderate to severe traumatic brain injury, in which the majority of head injured patients attend after mild mechanisms of injury. We recognize the following limitations of the study: in our population, we see a very low rate of moderate to severe TBI, therefore a case–control method was chosen. Cases were obtained from retrospective recruitment spanning 8 years – During this period there may have been changes to ED practices and protocols within the department. Also, we acknowledge that exaggerated results can trigger premature adoption of diagnostic tests [34]. In order that physicians make accurate informed decisions about the care for individual patients, larger prospective studies are required in a new population to validate these findings. We chose to perform age-matching in this study, to aid the ED physician when faced with a head-injured child of known age. We recognize, however, that matching by age would affect the independence of the observations, and that age could be associated with the other cofactors. This was not explored in this analysis. Given continued accrual of patients with moderate to severe head injury in the prospective database, we aim to take into account this aspect in the analysis. Finally, we recognize that the ML model in our study was built partially from statistically significant variables from logistic regression, and therefore the incorporation of variables for the two methods was not similar. The ML method serves to build on the logistic regression method as an improved tool, rather than a replacement of logistic regression. With a larger database, we will be able to validate this model on a separate dataset.

## Conclusions

In a population with a low event rate of moderate to severe TBI and a low CT rate, the following predictors were demonstrated to be significant in predicting moderate to severe TBI: road traffic accident as the mechanism of injury, a history of loss of consciousness, vomiting and signs of base of skull fracture. Moreover, seizure activity, confusion and clinical signs of skull fracture held predictive power in the diagnosis of pediatric TBI. In this study, we demonstrated the feasibility and the advantages of using machine learning as a tool to predict TBI. If validated on a large scale, the ML method has the potential not only to guide discretionary use of CT, but also a more careful selection of head injured children who warrant closer monitoring in the hospital.

## Abbreviations

AIG: Akaike criterion
AUC: Area under the ROC curve
BIC: Bayesian information criterion
CATCH: Canadian assessment of tomography for childhood head injury
CHALICE: Children’s head injury algorithm for the prediction of important clinical events
CDR: Clinical decision rules
CT: Computed tomography
ED: Emergency department
GCS: Glasgow coma scale
LOC: Loss of consciousness
ML: Machine learning
OR: Odds ratio
PECARN: Pediatric Emergency Care Applied Research Network
ROC: Receiver operating characteristic
TBI: Traumatic brain injury
